# Genome Size Doubling Arises From the Differential Repetitive DNA Dynamics in the Genus *Heloniopsis* (Melanthiaceae)

**DOI:** 10.3389/fgene.2021.726211

**Published:** 2021-09-06

**Authors:** Jaume Pellicer, Pol Fernández, Michael F. Fay, Ester Michálková, Ilia J. Leitch

**Affiliations:** ^1^Institut Botànic de Barcelona (IBB, CSIC-Ajuntament de Barcelona), Barcelona, Spain; ^2^Royal Botanic Gardens, Kew, Richmond, United Kingdom; ^3^School of Plant Biology, University of Western Australia, Crawley, WA, Australia; ^4^Department of Botany and Zoology, Faculty of Science, Masaryk University, Brno, Czechia

**Keywords:** *C*-value, DNA repeats, chromosome, transposable elements, satellite DNA

## Abstract

Plant genomes are highly diverse in size and repetitive DNA composition. In the absence of polyploidy, the dynamics of repetitive elements, which make up the bulk of the genome in many species, are the main drivers underpinning changes in genome size and the overall evolution of the genomic landscape. The advent of high-throughput sequencing technologies has enabled investigation of genome evolutionary dynamics beyond model plants to provide exciting new insights in species across the biodiversity of life. Here we analyze the evolution of repetitive DNA in two closely related species of *Heloniopsis* (Melanthiaceae), which despite having the same chromosome number differ nearly twofold in genome size [i.e., *H. umbellata* (1C = 4,680 Mb), and *H. koreana* (1C = 2,480 Mb)]. Low-coverage genome skimming and the RepeatExplorer2 pipeline were used to identify the main repeat families responsible for the significant differences in genome sizes. Patterns of repeat evolution were found to correlate with genome size with the main classes of transposable elements identified being twice as abundant in the larger genome of *H. umbellata* compared with *H. koreana*. In addition, among the satellite DNA families recovered, a single shared satellite (HeloSAT) was shown to have contributed significantly to the genome expansion of *H. umbellata*. Evolutionary changes in repetitive DNA composition and genome size indicate that the differences in genome size between these species have been underpinned by the activity of several distinct repeat lineages.

## Introduction

Plant genomes are dynamic and can expand in size through a variety of processes such as the proliferation of repetitive elements (including transposable elements and tandem repeats), and whole genome duplications (Wang D. et al., 2021). In parallel, regulatory mechanisms (i.e., epigenetic modifications) can act to prevent repetitive sequences from uncontrolled expansion and these, together with various recombination-based processes which may eliminate DNA, can result in genome downsizing (Devos et al., 2002; Hawkins et al., 2009; Schubert and Vu, 2016; Vu et al., 2017; Wang X. et al., 2021). It is now widely recognized that it is the relative activity of each of these opposing evolutionary forces driving genome expansion or contraction that has underpinned the generation of the outstanding diversity of genome sizes in plants, especially in angiosperms which vary c. 2,400-fold (Pellicer et al., 2018). Even at the genus level, genome size can vary by orders of magnitude, such as for example in *Cuscuta*, in which nuclear DNA content ranges c. 102-fold (Neumann et al., 2021). At the family level, Melanthiaceae stand out within monocots as being among the most diverse, with genome sizes varying > 230-fold (Pellicer et al., 2014). This is even more remarkable given that this family is made up of just c. 180 species, and similar levels of diversity have only been reported in much larger groups, such as the eudicot family Santalaceae (c. 1,000 species, 1C range: c. 395-fold).^1^ The main driver underpinning the extensive genome size diversity in Melanthiaceae lies in a striking genome expansion that occurred during the diversification of tribe Parideae. This event is estimated to have taken place c. 57-31 million years ago (Kim et al., 2019), and resulted in the emergence of some of the largest genomes known to date (Pellicer et al., 2014). Besides, a thorough analysis at lower taxonomic levels beyond Parideae revealed an almost doubling of the nuclear DNA content between species in *Heloniopsis*, a small genus made up of six species, with 1C-values ranging from 2,480 Mb in *H. koreana* to 4,680 Mb in *H. umbellata* (ratio = c. 1.90). This raises the question as to what are the key mechanisms responsible for underpinning such genome size differences among closely related taxa?

Although polyploidy is known to be frequent in many angiosperms and has indeed been reported in some genera of Melanthiaceae (e.g., *Paris*, *Trillium* and *Veratrum*), extant species of *Heloniopsis* share a chromosome number of 2n = 34 (Pellicer et al., 2014). The authors consistently found the same chromosome number across the tribe Heloniadeae, despite chromosome-based modeling approaches inferring with high probability that ancient polyploid events coupled with chromosome losses to have happened during the evolution of the tribe. Should this reconstructed scenario hold true, then it would imply that potential chromosomal reorganizations had not resulted in changes in the overall chromosome number or ploidy level, given the relatively stable karyotype features reported for several species in the genus (Kokubugata et al., 2004).

Considering the evolutionary past of the tribe, the observed differences in genome size can most be likely attributed to the differential activity of repetitive DNA sequences and the associated recombination-based mechanisms in charge of their removal. Repetitive DNA in plants includes both dispersed mobile elements and tandem repeats (Bennetzen and Wang, 2014). DNA transposon and retrotransposon dynamics involve cut-and-paste and copy-and-paste insertion mechanisms, respectively, to spread across the genome, and are recognized as dispersed mobile elements. Of these, long terminal repeat (LTR) retrotransposons are widely known to monopolize a substantial fraction of plant genomes, and comprise several superfamilies, with Ty1/copia and Ty3/gypsy elements being the most common in plants (Wicker et al., 2007). Indeed, in many cases, their dominance leaves a secondary role for tandem repeats in shaping plant genome evolutionary dynamics. This is the case, in for example, *Hesperis* (Brassicaceaee), where most of the genome size variation observed is driven by the activity of a diverse array of LTR families (Hloušková et al., 2019).

As our understanding of how genomes are structured and function increases, it is becoming apparent that at the lower end of the genome size spectrum, changes in size are often seen to be driven by the activity of just a few lineages of transposable elements (Hawkins et al., 2006; Piegu et al., 2006; Macas et al., 2015), whereas plants with larger genomes (i.e., > c. 10 Gb/1C) have most likely arisen through the accumulation of elements over long periods of time, given their more heterogeneous composition (Nystedt et al., 2013; Kelly et al., 2015; Novák et al., 2020a). Despite the above-mentioned critical role of transposable elements in shaping many plant genomes, Ågren et al. (2015) emphasized the need to also recognize the importance of short simple sequence repeats (including simple repeats, satellite and low complexity DNA) in contributing to the evolution of genome size in some plant species, using the evening primroses (*Oenothera* species) to illustrate this. Altogether, research aiming to uncover what variables influence the dynamics of repetitive DNA sequences in non-model plants, why they accumulate in some lineages and not others, and what are the key sequences involved, is urgently needed to continue to further our understanding of the origins of the staggering genome size diversity across eukaryotes in general, and in plants in particular.

To contribute to these goals, we have carried out a comparative study of two species of the genus *Heloniopsis* with contrasting genome sizes. We have used next generation sequencing to characterize and assess the abundance of different types of DNA repeats and their role in contributing to changes in the composition and size of both genomes. We also explore whether the differences in genome size between these two species are underpinned by (i) differences in the amounts of just a few repetitive elements, as observed in other species with small genomes, and (ii), evaluate the differences across the repetitive landscape composition between both species.

## Materials and Methods

### Plant Material and DNA Sequencing

Details for provenance and vouchers of *H. koreana* and *H. umbellata* can be found in Pellicer et al. (2014). These species were selected as they show the largest difference in genome size between the six species which comprise this genus, with *H. umbellata* (4,680 Mb/1C) having nearly double the DNA amount compared with *H. koreana* (2,480 Mb Mb/1C), and represent species belonging to the two main clades of this small genus. Genomic DNA extraction was carried out using the 2x CTAB method with minor modifications (Doyle and Doyle, 1987) followed by a CsCl/ethidium bromide density gradient and dialysis. The DNA products were run on an 1% agarose gel and quality control assessed using a Qubit 3 fluorometer (Thermo Fisher Scientific). Paired-end shotgun libraries with an average insert size of 500 bp were prepared and sequenced by Beijing Genomics Institute (BGI, Shenzhen, China) on an Illumina HiSeq 2000 (Illumina, San Diego, CA, United States) generating 100 nucleotide reads (0.15 × genome coverage). The quality of sequencing data was assessed using FastQC^2^ and reads were pre-processed using the FASTX-Toolkit.^3^ Sequence reads were filtered using a threshold quality score of 20 over the full length of the read. Reads of organellar origin were filtered using custom databases of monocot plastid and mitochondrial genomes (all available from NCBI at the time of analysis) using the standalone version of BLAST (v2.2.16; Altschul et al., 1997). Reads with significant hits to either the plastid or mitochondrial databases were then filtered using a custom Perl script (supplied by Laura J. Kelly). The remaining reads were thus considered to be of nuclear origin.

### Graph-Based Clustering in RepeatExplorer 2

Repeat identification by similarity-based clustering of Illumina paired-end reads was carried out using the Repeat Explorer 2 pipeline (Novák et al., 2013, 2020b), a GALAXY-based server for characterisation of repetitive elements.^4^ FASTQ reads were converted to FASTA format and interlaced prior to the clustering analysis. A preliminary round of clustering was performed with the original datasets [*H. koreana* = 3,127,826 reads (0.11×) and *H. umbellata* = 5,968,792 reads (0.11×)] to determine the maximum number of reads for each species to include in the final analysis. This employed the default settings (90% similarity over 55% of the read length, and cluster size threshold = 0.01%). After the initial screening, each set of reads was randomly down-sampled according to their genome size to represent reads comprising 1.5% of the genome of each species (i.e., genome proportion = 0.015×, *H. koreana* = 410,000 reads and *H. umbellata* = 784,993 reads). Automated repeat classification was based on connection-based clustering via paired-end reads and BLAST (n, x) similarity searches to REXdb (Neumann et al., 2019), a comprehensive database of conserved protein domains in retrotransposons. Output directories were individually examined for a final manual annotation and quantification of clusters and connections to superclusters. In addition, a comparative clustering analysis was carried out using a combined dataset of 1,015,000 reads (each species at a genome proportion equal to 0.013×). A four-letter prefix identity code was added to each sample dataset and used as the input to Repeat Explorer as described above. Repeat annotation of shared clusters between the two species was done following the same parameters as for the individual analyses.

### Genome Dynamics and Relative Abundance of Particular Repetitive Elements

The different repeat families in the two *Heloniopsis* species were recorded using the annotation output files from the Repeat Explorer analysis and summarized accordingly. Baseline statistics including, genome proportion (in percentage), abundance (Mb/1C), ratios of transposable elements and correlations between the main families of DNA repeats identified were calculated using R (R Core Team, 2019). A pairwise scatterplot of the main repeat element classes identified was constructed by comparing the number of shared reads between the two species based on McCann et al. (2018), and using the function *ggplot* built in the ggplot2 package (Wickham, 2016). The number of shared reads per cluster were obtained from the output files of the comparative analysis in Repeat Explorer. The slope of the line in the scatterplot represents the genome size ratio between the two species, thus any deviation from the line indicates biases in the contribution of a given element to the genome size of one species, compared with the other. Note that due to the large amounts of satellite DNA in the genome of *H. umbellata* compared with *H. koreana* this repeat type was not included in the scatterplot to enable a better visualization of the remaining data (but included in subsequent statistical regression analyses). Further linear model regression analyses of shared read clusters from the comparative Repeat Explorer analysis were carried out using the function *lm* in R *Stats* package (R Core Team, 2019).

To compare repeat abundances with changes in genome size, ancestral 1C-values in tribe Heloniadeae were reconstructed using maximum likelihood (ML) under a Brownian motion model using functions *ace* and *fastAnc* of the library *Phytools* (Revell, 2012). Genome size data available for extant species in Pellicer et al. (2014) and the phylogenetic tree from Kim et al. (2016) were used for the reconstruction. Following Macas et al. (2015), we also assessed the abundance of solo-LTRs, a product of ectopic unequal homologous recombination between LTRs of the same element type, in the two most abundant retrotransposon lineages (i.e., Ty1/copia-Angela and Ty3/gypsy-Tekay). Whilst not being conclusive, this approach provides an insight into the activity of one of the mechanisms by which LTR retrotransposons can be deleted from the genome (Cossu et al., 2017), and so can be used as a proxy to evaluate the potential impact of this process on genome size. Briefly, the method uses short Illumina reads to calculate an Rsf value, which is the ratio between the number of solo-LTRs to full-length elements for a particular repeat type. Larger Rsf values can indicate a higher impact of unequal homologous recombination. The analysis consists of five-steps: (1) Identification of the LTR-3′end and 5′-UTR junctions from read assemblies produced by the Repeat Explorer pipeline. (2) Extraction of 30 nt sequence tags which are used to create BLAST databases for the LTR-3′, 5′-UTR and a combined LTR-3′ + 5′-UTR (60 nt) regions. (3) BLAST all read sequences to the tag databases for the LTR-3′. (4) Blast all hits from the previous step against the 5′-UTR database. Finally, (5) BLAST hits from the previous step against the combined LTR-3′ + 5′-UTR database. These steps result in sets of reads representing LTR-3′end/5′-UTR junctions (LU) and LTR-3′end only (Lx). The Rsf ratio is then calculated using the formula: Rsf = (Lx—LU)/LU.

### Chromosome Preparations and Mapping of DNA Satellite HeloSAT by Fluorescence *in situ* Hybridisation (FISH)

Roots were collected from the same accessions used for genome size estimations and sequencing based on Pellicer et al. (2014). Briefly, roots were pre-treated in a saturated solution of 1-bromonaphthalene at 20°C for 24 h. Samples were then transferred to ice-cold 90% acetic acid for 10 min and stored in 70% ethanol at −20°C. Protoplast preparation was based on Kato et al. (2011). Roots were washed 3 × in ice-cold 1× citric buffer (50 mM sodium citrate, 50 mM EDTA, pH 5.5), then the tips were excised and macerated in 200 μL tubes containing 20 μL of enzymatic solution containing 4% cellulase Onozuka R-10 (Duchefa, Haarlem, The Netherlands) and 1% pectolyase from *Aspergillus niger* (Merck, Darmstadt, Germany) in 1× citric buffer pH 5.5 for 45–48 min at 37°C and transferred to ice. Digested roots were subsequently washed three times in ice cold 70% ethanol. Finally, 30 μL of ice-cold glacial acetic acid was added and mixed before dropping 4 μL of the protoplast suspension onto a microscope slide in a humid chamber until dry.

A non-denaturing and formamide-free fluorescent *in situ* hybridisation (FISH) protocol based on Cuadrado et al. (2009) and Mian (2019) was applied. A 26 bp oligo probe of HeloSAT was synthesized and labeled with FITC based on the output RE cluster monomers obtained (Supplementary Online Resource 1). The probe was evaluated to avoid self-hybridisation (i.e., dimerization and hairpins) with Oligo Calc.^5^ Oligo probes are single-stranded, therefore they do not need denaturation prior to hybridisation. The hybridisation mixture was simply prepared by diluting 2 μL of the 5′ end-labeled HeloSAT oligo (1 pmol/μL, Eurofins) in 1 × SSC pH 7.0 in a final volume of 15 μL. Hybridisation was carried out for 1 h at 37°C in a humid chamber. A post hybridization stringency wash was performed by transferring the slides to 1 × SSC 0.1% Triton X-100 buffer at 37°C, for long enough to allow coverslips to fall away from the slides (c. 5 min). The slides were then dehydrated in an ethanol series of 70, 90, and 100%, air-dried and subsequently counterstained with DAPI (Vectashield, Vector Laboratories, Burlingame, CA, United States). Preparations were examined using a Zeiss Axio Imager.Z2 fitted with an Axiocam 506 mono camera. Images were processed with Zeiss Zen 2.6 (blue edition) software (Zeiss).

## Results

### Repeat Content in *H. koreana* and *H. umbellata*

**FIGURE 1:**
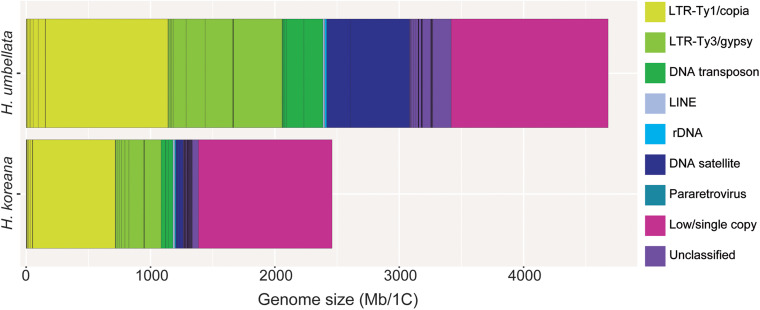
Genomic composition of *Heloniopsis koreana* (2,480 Mb/1C) and *H. umbellata* (4,680 Mb/1C). Estimates of the genomic abundance (in Mb/1C) of different repeats are colored by repeat class. The size of the unclassified (pale purple) and low/single copy fraction (pink) of each genome is also shown.

Details on the number of reads analyzed for each species and their genomic coverages are given in Table 1. A minimum coverage of 0.01% was required to classify a given cluster as repetitive DNA (i.e., a medium to high abundance repeat). The proportion of the genome estimated to be comprised of repetitive DNA sequences varied from 56.32% in *H. koreana* to 73.03% in *H. umbellata* (Table 2). Annotation and classification of the most abundant repeat clusters is presented in Table 2 and shown graphically in Figure 1. The clusters that failed to match any known elements from the REXdb where left as unclassified (i.e., 5.06% in *H. koreana* and 7.19% *H. umbellata*, Table 2). Overall, most of the identified repeats in the analysis of each species independently were more abundant in the larger genome of *H. umbellata*, with the exception of three specific lineages (i.e., Ty1/copia-Angela, Ty3/gypsy-Athila and LINE), which each had a higher genome proportion (in %) in the genome of *H. koreana* (Table 2). The repetitive landscape of both species was dominated by long terminal repeat (LTR) retrotransposons, which accounted for c. 60–78% of all identifiable DNA repeats. Among them, Ty1/copia-like elements were abundant, accounting for 29.04% (i.e., 714.45 Mb) and 24.27% (i.e., 1,134.43 Mb) of the genome in *H. koreana* and *H. umbellata*, respectively. Of these, Angela elements played a significant role in shaping these genomes, with genome proportions reaching 26.92% (i.e., 662.23 Mb) and 21.06% (i.e., 985.70 Mb) in *H. koreana* and *H. umbellata*, respectively. Other Ty1/copia lineages were present, but with a much lower contribution to each genome (i.e., <2%, Table 2 and Figure 1). Ty3/gypsy-like elements were also present in both species and accounted for a lower but still significant proportion of the genomes compared with Ty1/copia-like elements [i.e., 15.12% (i.e., 373.62 Mb) and 19.61% (917.79 Mb) of the repetitive fraction in *H. koreana* and *H. umbellata*, respectively]. The abundance of tandem repeats, namely satellite DNA, differed considerably between the species. While four different major satellite clusters were found in *H. koreana* (2.33%), in *H. umbellata*, only two satellite clusters were recovered, but these accounted for 14.24% of the genome (one of them having a genome proportion of 10.20%).

**TABLE 1:** Estimation of genome size and sequencing of the two *Heloniopsis* species studied.

**Species**	**Genome size 1C (Mbp)**	**Chrom number (2n)**	**Sequencing run number**	**Individual clustering**		**Comparative clustering**
				**No. reads**	**Coverage (×1C)**	**No. reads (0.013×)**
*H. koreana*	2,480	34	SRR15208643	410,000	0.015×	349,706
*H. umbellata*	4,680	34	SRR15208642	784,994	0.015×	665,294

**TABLE 2:** Repeat composition inferred in the studied *Heloniopsis* species.

**Repeat type**	**Lineage**	**Genome proportion (%)**	
		***H. koreana***	***H. umbellata***
**Retrotransposon**			
Ty1/copia			
	**All**	**29.04**	**24.27**
	Angela	26.92	21.06
	Ale	0.85	1.63
	TAR	0.87	0.90
	Tork	0.29	0.43
	Ikeros	0.10	0.23
Ty3/gypsy			
	**All**	**15.12**	**19.61**
	Retand	6.04	8.80
	Tekay	2.18	4.80
	Athila	5.07	3.52
	Tat/Ogre	1.84	2.23
	CRM	0.06	0.25
Other repeats			
	LINE	0.61	0.30
	Pararetrovirus	–	0.01
**DNA transposons**			
	**All**	**3.85**	**7.02**
	Enspm/CACTA	2.96	6.26
	hAT	0.83	0.51
	MuDR	0.06	0.17
	Helitron	–	0.08
**Tandem repeats**			
	rDNA	0.24	0.36
	Satellite	2.33	14.24
Unclassified		5.06	7.19
Total repeats		**56.32**	**73.03**
Low/single copy		43.67	26.97

*Bold values are refer to the cumulative overall proportions of elements belonging to the same group.*

### Genome Size and Comparative Repeat Dynamics in *Heloniopsis*

**FIGURE 2:**
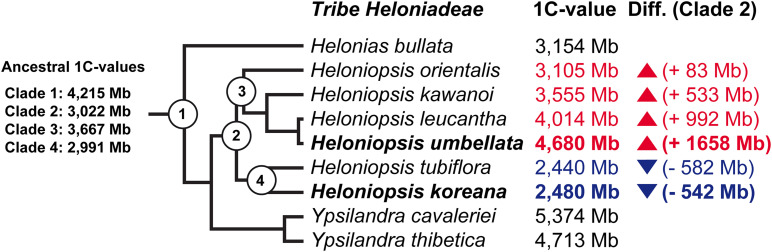
Summary of the ancestral genome size reconstruction in tribe Heloniadeae. Branch tips include the extant 1C-values taken from Pellicer et al. (2014) with the increase (red) or reduction (blue) in genome size indicated based on the 1C-value reconstructed for the most recent common ancestor of the genus (i.e., Clade 2).

**FIGURE 3:**
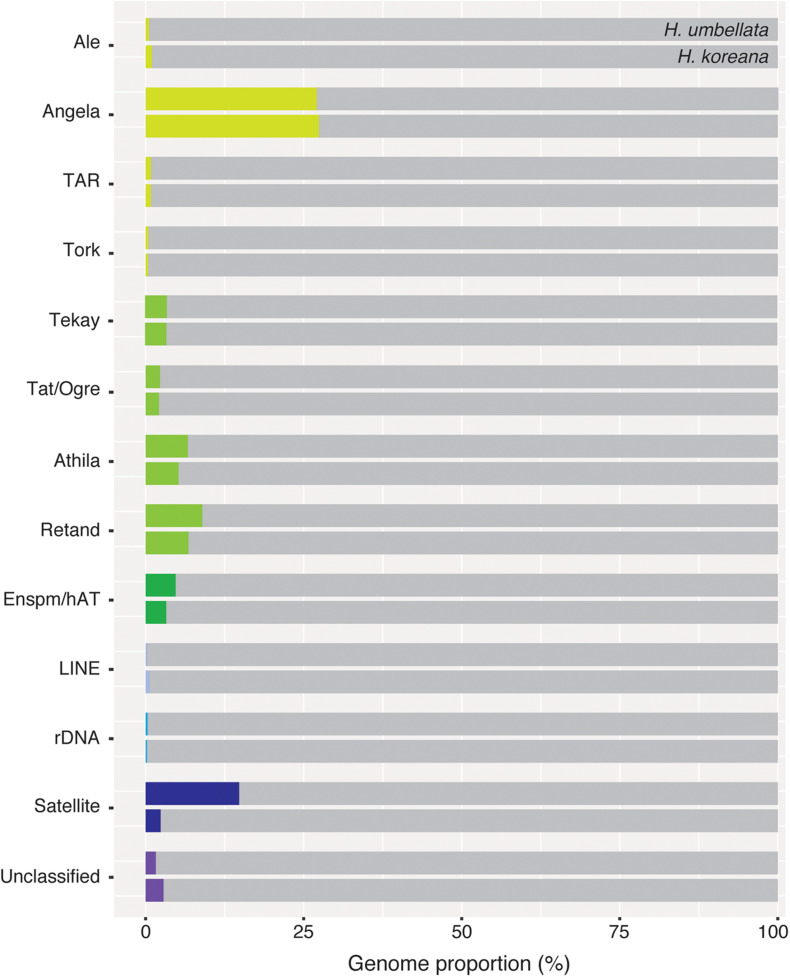
Genomic proportions (in %) of shared repetitive elements between *Heloniopsis koreana* and *H. umbellata*.

**FIGURE 4:**
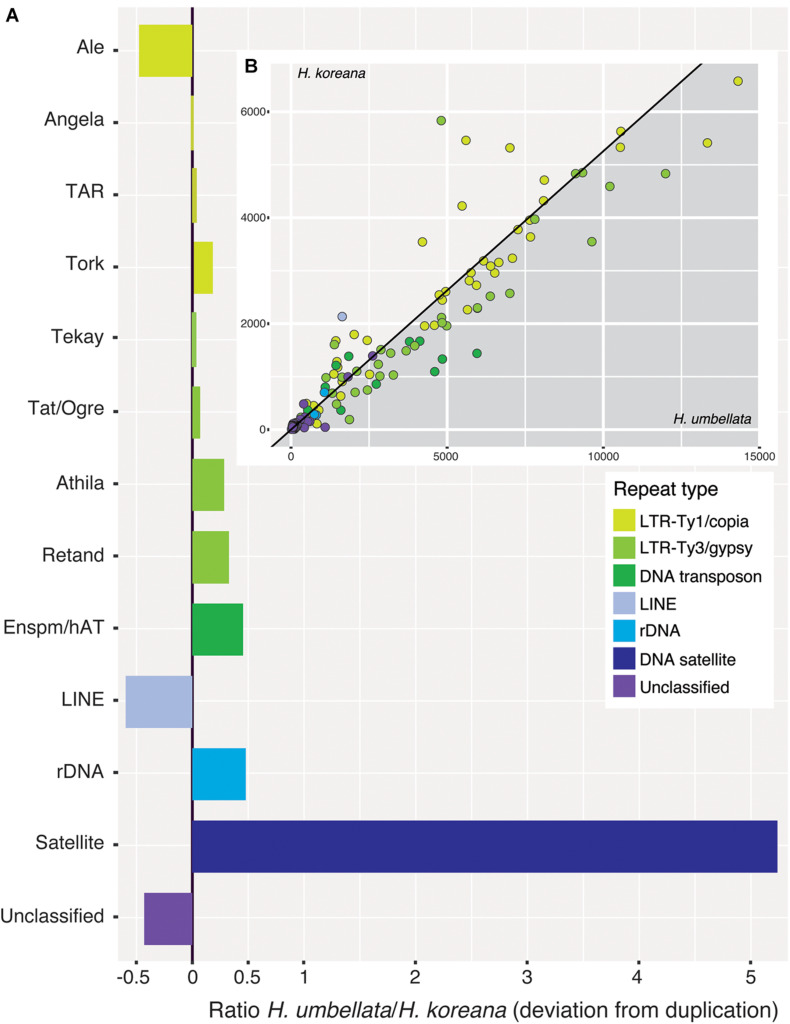
**(A)** Ratios of incidence of shared repeat clusters from the comparative analysis (*Heloniopsis umbellata*/*H. koreana*). A ratio of zero indicates clusters present in the same genomic proportion in both species. **(B)** (inset) Pairwise scatterplot of the number of reads from each species in shared repeat clusters from the comparative analysis (excluding satellite DNA). The slope of the line is equal to the ratio of the genome sizes of the two species (i.e., 1.9). Dots falling along the line are present in the same genomic proportions in the two species.

Ancestral 1C-values (Anc1C) reconstruction in Heloniadeae is depicted in Figure 2. The most recent common ancestor (MRCA) of *Heloniopsis* was reconstructed to have an Anc1C = 3,022 Mb (Figure 2, clade 2). Since then, contrasting genome size dynamics have been inferred in the two main clades of the genus (Figure 2, clades 3 and 4), illustrating that ups and downs in genome size have taken place during the evolution of the genus. The comparative clustering involved analyzing 1.05 million reads (0.013 × GP/1C per species). The genomic proportions (in %) of the shared top 20 superclusters (grouped by repeat classification) are depicted in Figure 3. The ratios observed between the genome proportions of these shared repeats in each species are illustrated in Figure 4A, and show that a few repeat types (e.g., Ty1/copia-Angela, Ty1/copia-TAR, Ty3/gypsy-Tekay) occur in similar genomic proportions in the two species and hence indicating that they are (nearly) twice as abundant in total copy number in *H. umbellata* compared with *H. koreana*. Nevertheless, other repeat types deviate from this ratio, and hence comprise a higher (e.g., Ty1/copia-Ale) or lower (e.g., Ty3/gypsy-Athila) genome proportions in *H. koreana* compared with *H. umbellata*. At the sequence read level, by comparing the total number of reads from each species in each repeat cluster, this trend is also illustrated in Figure 4B, where many reads in shared clusters were biased toward contributing to the genome of *H. umbellata* (especially clusters containing over 2,500 reads).

Table 3 shows the regressions between the abundance of different repeat types based on the genome sizes of the two *Heloniopsis* species studied. Significant strong relationships were found based on the genome sizes and the two major lineages of retrotransposons which have the highest impact on the genome composition of these two species [i.e., Ty1/copia-Angela (*R*^2^ = 0.76, *p* = 1.32e^–09^) and Ty3/gypsy-Retand (*R*^2^ = 0.96, *p* = 4.28e^–08^)]. A positive correlation was also found when all the LTR-retrotransposon elements were analyzed together (*R*^2^ = 0.81, *p* = 2.01e^–16^). However, when all DNA repeats were analyzed as a whole, DNA satellites were seen to have a significant impact on the regression, as shown by the improvement of the correlation coefficient when DNA satellites were excluded from the analysis (*R*^2^ = 0.26, *p* = 3.49e^–11^ versus *R*^2^ = 0.88, *p* = 2.24e^–16^, respectively).

**TABLE 3:** Linear model regressions of repeat contents with genome size variation among *H. koreana* and *H. umbellata*.

**Repeats (*H. koreana/ H. umbellate)***	***R* ^2^**	***p*-value**
All LTR-retrotransposons	0.818	2.01e^–16^
Ty1/copia*-*Angela	0.762	1.32e^–09^
Ty3/gypsy*-*Retand	0.969	4.28e^–08^
All repeats		
(including DNA satellites)	0.259	3.49e^–11^
(excluding DNA satellites)	0.875	2.24e^–16^

To explore whether differences in the amount of unequal homologous recombination between the LTR sequences could be contributing to the differences in genome size between the two *Heloniopsis* species, we identified the LTR-3′ end and 5′ untranslated regions from the Ty1/copia-Angela and Ty3/gypsy-Retand elements, which were the two most abundant transposable elements (Table 2). Despite the relatively high Rsf values estimated for both repeats in both species, and the caution that needs to be paid when interpreting these data as evidence for recombination using this approach, the values obtained for *H. koreana* (Rsf-Angela: 11.41, Rsf-Retand: 4.22), were higher than those in *H. umbellata* (Rsf-Angela: 7.94, Rsf-Retand: 3.88).

### Identification and Characterisation of DNA Satellites

The clustering analysis identified four distinct types of satellite DNA, two of which were specific to the *H. koreana* genome while the other two were present in both species (Supplementary Online Resource 1). The abundance of the two shared DNA satellites varied between species, particularly in *H. umbellata* where their combined abundance was over six times higher (i.e., 14.24%) than in *H. koreana* (2.33%, Figure 4A). The abundance of just one of these satellites in particular, hereafter named HeloSAT, highlights the contrasting evolutionary dynamics between these two closely related species. A single cluster of HeloSAT accounted for 10.20% of the genome in *H. umbellata*, while comprising just 0.83% in *H. koreana*.

To further investigate the presence of this satellite from a comparative viewpoint, HeloSAT was physically mapped onto the chromosomes and interphase nuclei of both species using fluorescent *in situ* hybridization (FISH) (Supplementary Online Resource 2). Results from FISH corroborated those from the clustering approach, with HeloSAT signals being more abundant and spread across the chromosomes of *H. umbellata* (Supplementary Online Resource 2A) than in *H. koreana* (Supplementary Online Resource 2C). The size, fluorescence intensity and number of signals in the latter were lower, which was even more evident when analyzing interphase nuclei from both species (Supplementary Online Resource 2B,D).

## Discussion

### Diversity and Dynamics of Repetitive Elements in *Heloniopsis*

In this work we provide the first insights into genome evolutionary dynamics in the genus *Heloniopsis*, by combining high throughput sequence data and cytogenetics. Genome size evolution in the genus may be considered to be bi-directional based on our ancestral state reconstruction analysis, which showed opposite evolutionary trajectories in the two clades (Figure 2). Our analysis showed that the most recent common ancestor of *Heloniopsis* likely had an Anc1C of 3,022 Mb, indicating that during the evolution of *H. umbellata* its genome has expanded by 1,658 Mb. Such a trend is in striking contrast to that observed in *H. koreana*, in which we inferred a genome reduction of 542 Mb with respect to the MCRA of the genus. Based on the study by Kim et al. (2019), genome size divergence is estimated to have taken place within a c. 10 Mya period, possibly as far back as the Miocene, when the genus is estimated to have started to diverge. The observed genome sizes, however, do not preclude the possibility that additional shifts also took place during the evolution of the genus, thus our analyses should therefore be seen as just one potential evolutionary scenario based on genome size data from extant species. Certainly, shifts in genome size during the evolutionary history of plants have been reported in many plant lineages (e.g., Lysak et al., 2009; Pellicer et al., 2013; Vallès et al., 2013; Kelly et al., 2015), and Melanthiaceae are no exception. Furthermore, the fact that both species show relatively high proportions of solo-LTRs (i.e., Rsf values) indicate that despite recombination likely affecting *H. koreana* more significantly than *H. umbellata*, based on the higher Rsf values in the two most abundant repeats (i.e., Ty1/copia Angela and Ty3/gypsy-Retand), both species appear to have reduced the abundance of these major repeat types contributing to the overall genome size of these species, and this may have led to an overall genomic contraction if such recombination processes have been sufficiently active to overcome the impact of repeat amplification.

In the absence of polyploidy, the dynamics of transposable elements (mainly LTR-retrotransposons belonging to Ty1/copia and Ty3/gypsy superfamilies) underpin most changes in genome size given their significant contribution to the genomic landscape in plants (e.g., McCann et al., 2020). The repetitive fraction of the *Heloniopsis* genomes explored here include several transposable elements and tandem repeats, and the genome size differences between the two species studied can be explained, at least partially, by their repetitive content, given the significant overall correlation between their abundance and genome size variation (Table 3). The individual clustering analysis revealed that 56.3% of the genome of *H. koreana* is highly repetitive, whereas in *H. umbellata*, with a larger genome, the repetitive fraction reached 73% of its genome. Such proportions fall within the ranges previously reported for seed plants with similar genome size to those investigated here (Novák et al., 2020a), with the differences in the repetitive landscape observed here providing support for the contrasting genome sizes between the two species analyzed. Indeed, the larger genome of *H. umbellata* containing a larger proportion of repeats, many of which occupied a similar genome proportion to *H. koreana* indicate that their copy number has nearly doubled (Figure 4).

The detailed characterisation and identification of repetitive DNA content in the two *Heloniopsis* species studied highlighted the relative impact of the Ty1/copia-Angela elements in their genomes, with proportions ranging from c. 21 to 26% (Table 2). Among the Ty1/copia lineages that have been identified in plants (Neumann et al., 2019), Angela elements have been reported to be abundant in other genomes, with similar proportions as found here (e.g., *Passiflora*, *Thinopyrum*; Divashuk et al., 2020; Sader et al., 2021). Such proportions are, nonetheless, lower than for other LTR-retrotransposon elements reported in some plant genomes of comparable size to those of *Heloniopsis*. For example, Tekay/Del elements which belong to the Ty3/gypsy lineage were reported to account for c. 67 and 97% of the repetitive landscape in *Capsicum anuum* and *C. chinense*, respectively (de Assis et al., 2020), although they only accounted for 15% of the repetitive genome of the closely related *C. baccatum* (of similar genome size), illustrating that even within a genus, contrasting evolutionary dynamics can give rise to distinctive repeat profiles. Overall, the analyses indicate that it is often the combined activity of a diverse array of repeat lineages which contribute to differences in genome size observed between species rather than the differential rates of amplification/deletion of just one or few transposable element families. Despite this, our observations on the clustering analysis also suggest that transpositional bursts might have occurred, as in the case of Ty1/copia-Angela, where large superclusters were recovered (Figure 1). This would result in higher levels of homogeneity between sequence copies, a pattern in direct contrast to evidence from much larger genomes, such as in those of *Fritillaria* in which even the smallest genome analyzed (in *F. davidii*, 33,525 Mb/1C) is over seven times larger than *H. umbellata* (Kelly et al., 2015). The repeat composition of these immense genomes is highly heterogeneous, indicative of long-term amplification processes combined with low rates of deletion, resulting in a wealth of relatively low−abundance repeat−derived DNAs. Furthermore, retrotransposition can occur at different rates, even between closely related species, making it sometimes difficult to interpret repetitive DNA composition and dynamics in relation to genome size because of the challenges of uncovering the signatures of recombination-based mechanisms from short-read sequence data (e.g., Macas et al., 2015). Indeed, an analysis of genome diversity in *Anacyclus* (Asteraceae), revealed that changes in genome size were more significantly underpinned by chromosomal restructuring than by differential dynamics of a reduced set of high-copy-number transposable element families (Vitales et al., 2020).

### The Impact of Satellite DNA in Shaping Genome Evolution of *Heloniopsis*

Despite LTR-retrotansposons being the most abundant repeat types uncovered in many plant genomes, the analysis of tandem repeats (i.e., satellite DNA) has also revealed a great diversity in terms of sequence composition, organization and genomic abundance across different land plant species (Garrido-Ramos, 2015). In *Heloniopsis*, compared with other types of repeats identified (see above), satellite DNAs are not the major genomic component. Nevertheless, differences in their abundance have contributed to the differences in genome size observed between the two species analyzed. This is shown by the contrasting genome proportions of the most abundant satellite identified called HeloSAT. Thus, although HeloSAT accounted for up to c. 477 Mb (i.e., 10.20%) of the *H. umbellata* genome, its genome proportion in *H. koreana* was just c. 0.83%. To further explore this satellite repeat, its overall physical organization along the chromosomes of the two *Heloniopsis* species was determined using FISH. As Supplementary Online Resource 2 shows, there were more hybridisation signals visible on the chromosomes and interphase nuclei of *H. umbellata* than of *H. koreana*. Although FISH is not a fully quantitative technique, the results support the contrasting genome proportions of this satellite in *H. umbellata* compared with *H. koreana* estimated using Repeat Explorer. The satellite appeared to be more widely distributed across the genome of *H. umbellata*, than that of *H. koreana*, with hybridisation signals present on most chromosomes. Nevertheless, despite genome size and satellite size correlating to some extent in *Heloniopsis*, such a trend is not the rule across all plant lineages studied to date. For example, closely related *Paphiopedilum* species with very similar genome sizes were shown to contain divergent satellite elements which differed considerably in abundance between closely related species (Lee et al., 2018). The data provided support to the suggestion that satellite DNAs often evolve rapidly and differ considerably in abundance even in related species with little correlation with genome size (Macas et al., 2010). In addition, recent research in *Passiflora* (1C range = 207.34–2,621.04 Mb) reported an unusually large number of satellite repeats in the species with the smallest genome, albeit at lower frequencies, leading the authors to propose that, in most species, tandem repeats have only a limited impact on the overall genome size of *Passiflora* (Sader et al., 2021).

The number and types of satellite DNAs present in plant genomes can be highly variable. For instance, in *Cuscuta*, > 113 putative DNA satellites were recovered, with relatively substantial genome proportions up to c. 15–18%, and comprising several gigabasepairs in some taxa with relatively large genomes (i.e., 3,400 Mbp/1C) (Neumann et al., 2021). Similarly, in *Vicia peregrina*, 51 satellites were identified (Macas et al., 2015), whereas in *Luzula elegans* 37 satellites were reported (Heckmann et al., 2013). In contrast, other species including *Heloniopsis* have been shown to have a much lower diversity of satellite types, and a higher incidence of species-specific satellites (e.g., Macas et al., 2011; Lee et al., 2018; Mata-Sucre et al., 2020). This is the case, for example in *Fritillaria affinis*—in which only one satellite—FriSAT—was identified, although it accounted for c. 11% of its genome, and it was almost absent in closely related species (Kelly et al., 2015), indicative of rapidly evolving DNA clusters with strong phylogenetic signal.

## Conclusion

Novel data characterizing the repetitive DNA landscape in *Heloniopsis* have been presented using genome skimming data from short read high throughput sequencing. Although polyploidy and the differential activity of repetitive DNAs have been shown to be major drivers of genome size evolution in plants, even in some closely related species, differences in genome size may evolve through contrasting repeat dynamics alone. Our analysis of the repetitive genome of two *Heloniopsis* species illustrates the latter, as the nearly twofold difference in genome size between species has arisen without any change in chromosome number. The detailed characterisation and comparative analysis of the repetitive DNA content of *H. umbellata* and *H. koreana* show that their genomes are dominated by LTR-retrotransposons, with the larger genome of *H. umbellata* mainly being determined by the increased abundance of the same LTR-retrotransposon elements already present in *H. koreana* rather than the amplification of new repeat types. Few satellite DNAs were recovered, but the characterisation of HeloSAT and its abundance in the genome of *Heloniopsis*, especially *H. umbellata*, provides support for the relevance of satellite DNA in shaping genome size evolution in some plant species.

## Data Availability Statement

The datasets presented in this study can be found in online repositories. The names of the repository/repositories and accession number(s) can be found below: https://www.ncbi.nlm.nih.gov/, SRR15208642; https://www.ncbi.nlm.nih.gov/, SRR15208643.

## Author Contributions

JP, MFF, and IJL conceived and designed the study. JP, PF, and EM conducted the laboratory work and analyses of data. JP and IJL wrote the manuscript and all authors reviewed and edited the manuscript. All authors have read and agreed to the published version of the manuscript.

## Conflict of Interest

The authors declare that the research was conducted in the absence of any commercial or financial relationships that could be construed as a potential conflict of interest. The reviewer AK declared a past co-authorship with the authors JP, IJL to the handling editor.

## Publisher’s Note

All claims expressed in this article are solely those of the authors and do not necessarily represent those of their affiliated organizations, or those of the publisher, the editors and the reviewers. Any product that may be evaluated in this article, or claim that may be made by its manufacturer, is not guaranteed or endorsed by the publisher.

## Footnotes

1
https://cvalues.science.kew.org

2
http://www.bioinformatics.babraham.ac.uk/projects/fastqc/

3
http://hannonlab.cshl.edu/fastx_toolkit/

4
https://repeatexplorer-elixir.cerit-sc.cz/galaxy/

5
http://biotools.nubic.northwestern.edu/OligoCalc.html

## Supplementary Material

Supplementary Online Resource 1 Top four-most abundant monomers of HeloSAT.

Supplementary Online Resource 2 Physical mapping of the satellite DNA (HeloSAT) on chromosomes and interphase nuclei of *Heloniopsis umbellata* **(A,B)** and *H. koreana* **(C,D)**.

## References

[B1] Ågren J. A. Greiner S. Johnson M. T. J. Wright S. I. (2015). No evidence that sex and transposable elements drive genome size variation in evening primroses. *Evolution (N. Y.)* 69 1053–1062. 10.1111/evo.12627 25690700

[B2] Altschul S. F. Madden T. L. Schäffer A. A. Zhang J. Zhang Z. Miller W. (1997). Gapped BLAST and PSI-BLAST: a new generation of protein database search programs. *Nucleic Acids Res.* 25 3389–3402. 10.1093/nar/25.17.3389 9254694 PMC146917

[B3] Bennetzen J. L. Wang H. (2014). The contributions of transposable elements to the structure, function, and evolution of plant genomes. *Annu. Rev. Plant Biol.* 65 505–530. 10.1146/annurev-arplant-050213-035811 24579996

[B4] Cossu R. M. Casola C. Giacomello S. Vidalis A. Scofield D. G. Zuccolo A. (2017). LTR retrotransposons show low levels of unequal recombination and high rates of intraelement gene conversion in large plant genomes. *Genome Biol. Evol.* 9 3449–3462. 10.1093/gbe/evx260 29228262 PMC5751070

[B5] Cuadrado Á Golczyk H. Jouve N. (2009). A novel, simple and rapid nondenaturing FISH (ND-FISH) technique for the detection of plant telomeres. Potential used and possible target structures detected. *Chromosom. Res.* 17:755. 10.1007/s10577-009-9060-z 19669910

[B6] de Assis R. Baba V. Y. Cintra L. A. Gonçalves L. S. A. Rodrigues R. Vanzela A. L. L. (2020). Genome relationships and LTR-retrotransposon diversity in three cultivated *Capsicum* L. (Solanaceae) species. *BMC Genomics* 21:237. 10.1186/s12864-020-6618-9 32183698 PMC7076952

[B7] Devos K. M. Brown J. K. M. Bennetzen J. (2002). Genome size seduction through illegitimate recombination counteracts genome expansion in *Arabidopsis*. *Genome Res.* 12 1075–1079. 10.1101/gr.132102 12097344 PMC186626

[B8] Divashuk M. G. Karlov G. I. Kroupin P. Y. (2020). Copy number variation of transposable elements in *Thinopyrum* intermedium and its diploid relative species. *Plants* 9:15. 10.3390/plants9010015 31877707 PMC7020174

[B9] Doyle J. J. Doyle J. L. (1987). A rapid DNA isolation procedure for small quantities of fresh leaf tissue. *Phytochem. Bull.* 19 11–15.

[B10] Garrido-Ramos M. A. (2015). Satellite DNA in plants: more than just rubbish. *Cytogenet. Genome Res.* 146 153–170. 10.1159/000437008 26202574

[B11] Hawkins J. S. Kim H. Nason J. D. Wing R. A. Wendel J. F. (2006). Differential lineage-specific amplification of transposable elements is responsible for genome size variation in *Gossypium*. *Genome Res.* 16 1251–1261. 10.1101/gr.5282906 16954538 PMC1581434

[B12] Hawkins J. S. Proulx S. R. Rapp R. A. Wendel J. F. (2009). Rapid DNA loss as a counterbalance to genome expansion through retrotransposon proliferation in plants. *Proc. Natl. Acad. Sci. U.S.A.* 106 17811–17816. 10.1073/pnas.0904339106 19815511 PMC2764891

[B13] Heckmann S. Macas J. Kumke K. Fuchs J. Schubert V. Ma L. (2013). The holocentric species *Luzula elegans* shows interplay between centromere and large-scale genome organization. *Plant J.* 73 555–565. 10.1111/tpj.12054 23078243

[B14] Hloušková P. Mandáková T. Pouch M. Trávníček P. Lysak M. A. (2019). The large genome size variation in the *Hesperis* clade was shaped by the prevalent proliferation of DNA repeats and rarer genome downsizing. *Ann. Bot.* 124 103–120. 10.1093/aob/mcz036 31220201 PMC6676390

[B15] Kato A. Lamb J. C. Albert P. S. Danilova T. Han F. Gao Z. (2011). “Chromosome Painting for Plant Biotechnology,” in *Plant Chromosome Engineering: Methods and Protocols*, ed. Birchler J. A. (New York, NY: Springer), 67–96. 10.1007/978-1-61737-957-4_4 21181525

[B16] Kelly L. J. Renny-Byfield S. Pellicer J. Macas J. Novák P. Neumann P. (2015). Analysis of the giant genomes of *Fritillaria* (Liliaceae) indicates that a lack of DNA removal characterizes extreme expansions in genome size. *New Phytol.* 208 596–607. 10.1111/nph.13471 26061193 PMC4744688

[B17] Kim C. Kim S.-C. Kim J.-H. (2019). Historical biogeography of Melanthiaceae: a case of out-of-North America through the Bering land bridge. *Front. Plant Sci.* 10:396. 10.3389/fpls.2019.00396 31019522 PMC6458295

[B18] Kim S.-C. Kim J. S. Chase M. W. Fay M. F. Kim J.-H. (2016). Molecular phylogenetic relationships of Melanthiaceae (Liliales) based on plastid DNA sequences. *Bot. J. Linn. Soc.* 181 567–584. 10.1111/boj.12405

[B19] Kokubugata G. Peng C. I. Yokota M. (2004). Comparison of karyotypes among three *Heloniopsis* species from Ryuku Archipelago and Taiwan. *Ann. Tsukuba Bot. Gard.* 23 13–16.

[B20] Lee Y.-I. Yap J. W. Izan S. Leitch I. J. Fay M. F. Lee Y.-C. (2018). Satellite DNA in *Paphiopedilum* subgenus *Parvisepalum* as revealed by high-throughput sequencing and fluorescent in situ hybridization. *BMC Genomics* 19:578. 10.1186/s12864-018-4956-7 30068293 PMC6090851

[B21] Lysak M. A. Koch M. A. Beaulieu J. M. Meister A. Leitch I. J. (2009). The dynamic ups and downs of genome size evolution in Brassicaceae. *Mol. Biol. Evol.* 26 85–98. 10.1093/molbev/msn223 18842687

[B22] Macas J. Kejnovský E. Neumann P. Novák P. Koblížková A. Vyskot B. (2011). Next generation sequencing-based analysis of repetitive DNA in the model dioceous plant *Silene latifolia*. *PLoS One* 6:e27335. 10.1371/journal.pone.0027335 22096552 PMC3212565

[B23] Macas J. Neumann P. Novák P. Jiang J. (2010). Global sequence characterization of rice centromeric satellite based on oligomer frequency analysis in large-scale sequencing data. *Bioinformatics* 26 2101–2108. 20616383 10.1093/bioinformatics/btq343

[B24] Macas J. Novák P. Pellicer J. Čížková J. Koblížková A. Neumann P. (2015). In depth characterization of repetitive DNA in 23 plant genomes reveals sources of genome size variation in the legume tribe Fabeae. *PLoS One* 10:e0143424. 10.1371/journal.pone.0143424 26606051 PMC4659654

[B25] Mata-Sucre Y. Sader M. Van-Lume B. Gagnon E. Pedrosa-Harand A. Leitch I. J. (2020). How diverse is heterochromatin in the *Caesalpinia* group? Cytogenomic characterization of *Erythrostemon hughesii* Gagnon & G.P. Lewis (Leguminosae: Caesalpinioideae). *Planta* 252:49. 10.1007/s00425-020-03453-8 32918627

[B26] McCann J. Jang T.-S. Macas J. Schneeweiss G. M. Matzke N. J. Novák P. (2018). Dating the species network: allopolyploidy and repetitive DNA evolution in american saisies (*Melampodium sect*. *Melampodium*, Asteraceae). *Syst. Biol.* 67 1010–1024. 10.1093/sysbio/syy024 29562303 PMC6193527

[B27] McCann J. Macas J. Novák P. Stuessy T. F. Villaseñor J. L. Weiss-Schneeweiss H. (2020). Differential genome size and repetitive DNA evolution in diploid species of *Melampodium sect*. *Melampodium* (Asteraceae). *Front. Plant Sci.* 11:362. 10.3389/fpls.2020.00362 32296454 PMC7136903

[B28] Mian S. (2019). *The Impact of Genomic Structural Variation on Meiotic Pairing and Segregation in Beta vulgaris subsp. maritima*. Ph. D. thesis. London: Queen Mary University of London, 10.34885/61

[B29] Neumann P. Novák P. Hoštáková N. Macas J. (2019). Systematic survey of plant LTR-retrotransposons elucidates phylogenetic relationships of their polyprotein domains and provides a reference for element classification. *Mob. DNA* 10:1. 10.1186/s13100-018-0144-1 30622655 PMC6317226

[B30] Neumann P. Oliveira L. Čížková J. Jang T.-S. Klemme S. Novák P. (2021). Impact of parasitic lifestyle and different types of centromere organization on chromosome and genome evolution in the plant genus *Cuscuta*. *New Phytol.* 229 2365–2377. 10.1111/nph.17003 33090498

[B31] Novák P. Guignard M. S. Neumann P. Kelly L. J. Mlinarec J. Koblížková A. (2020a). Repeat-sequence turnover shifts fundamentally in species with large genomes. *Nat. Plants.* 6 1325–1329. 10.1038/s41477-020-00785-x 33077876

[B32] Novák P. Neumann P. Macas J. (2020b). Global analysis of repetitive DNA from unassembled sequence reads using RepeatExplorer2. *Nat. Protoc.* 15 3745–3776. 10.1038/s41596-020-0400-y 33097925

[B33] Novák P. Neumann P. Pech J. Steinhaisl J. Macas J. (2013). RepeatExplorer: a Galaxy-based web server for genome-wide characterization of eukaryotic repetitive elements from next-generation sequence reads. *Bioinformatics* 29 792–793. 10.1093/bioinformatics/btt054 23376349

[B34] Nystedt B. Street N. R. Wetterbom A. Zuccolo A. Lin Y.-C. Scofield D. G. (2013). The Norway spruce genome sequence and conifer genome evolution. *Nature* 497 579–584. 10.1038/nature12211 23698360

[B35] Pellicer J. Hidalgo O. Dodsworth S. Leitch I. J. (2018). Genome size diversity and its impact on the evolution of land plants. *Genes (Basel).* 9:88. 10.3390/genes9020088 29443885 PMC5852584

[B36] Pellicer J. Kelly L. J. Leitch I. J. Zomlefer W. B. Fay M. F. (2014). A universe of dwarfs and giants: genome size and chromosome evolution in the monocot family Melanthiaceae. *New Phytol.* 201 1484–1497. 10.1111/nph.12617 24299166

[B37] Pellicer J. Kelly L. J. Magdalena C. Leitch I. J. (2013). Insights into the dynamics of genome size and chromosome evolution in the early diverging angiosperm lineage Nymphaeales (water lilies). *Genome* 56 437–449. 10.1139/gen-2013-0039 24168627

[B38] Piegu B. Guyot R. Picault N. Roulin A. Sanyal A. Kim H. (2006). Doubling genome size without polyploidization: dynamics of retrotransposition-driven genomic expansions in *Oryza australiensis*, a wild relative of rice. *Genome Res.* 16 1262–1269. 10.1101/gr.5290206 16963705 PMC1581435

[B39] R Core Team (2019). *R: A Language and Environment for Statistical Computing.* Vienna: R Foundation for Statistical Computing.

[B40] Revell L. J. (2012). phytools: an R package for phylogenetic comparative biology (and other things). *Methods Ecol. Evol.* 3 217–223. 10.1111/j.2041-210X.2011.00169.x

[B41] Sader M. Vaio M. Cauz-Santos L. A. Dornelas M. C. Vieira M. L. C. Melo N. (2021). Large vs small genomes in *Passiflora*: the influence of the mobilome and the satellitome. *Planta* 253:86. 10.1007/s00425-021-03598-0 33792791

[B42] Schubert I. Vu G. T. H. (2016). Genome stability and evolution: attempting a holistic view. *Trends Plant Sci.* 21 749–757. 10.1016/j.tplants.2016.06.003 27427334

[B43] Vallès J. Canela M. Á Garcia S. Hidalgo O. Pellicer J. Sánchez-Jiménez I. (2013). Genome size variation and evolution in the family Asteraceae. *Caryologia* 66 221–235. 10.1080/00087114.2013.829690

[B44] Vitales D. Álvarez I. Garcia S. Hidalgo O. Nieto Feliner G. Pellicer J. (2020). Genome size variation at constant chromosome number is not correlated with repetitive DNA dynamism in *Anacyclus* (Asteraceae). *Ann. Bot.* 125 611–623. 10.1093/aob/mcz183 31697800 PMC7103019

[B45] Vu G. T. H. Cao H. X. Reiss B. Schubert I. (2017). Deletion-bias in DNA double-strand break repair differentially contributes to plant genome shrinkage. *New Phytol.* 214 1712–1721. 10.1111/nph.14490 28245065

[B46] Wang D. Zheng Z. Li Y. Hu H. Wang Z. Du X. (2021). Which factors contribute most to genome size variation within angiosperms? *Ecol. Evol.* 11 2660–2668. 10.1002/ece3.7222 33767827 PMC7981209

[B47] Wang X. Morton J. Pellicer J. Leitch I. J. Leitch A. R. (2021). Genome downsizing after polyploidy: mechanisms, rates and selection pressures. *Plant J.* 10.1111/tpj.15363 [Epub ahead of print]. 34077584

[B48] Wicker T. Sabot F. Hua-Van A. Bennetzen J. L. Capy P. Chalhoub B. (2007). A unified classification system for eukaryotic transposable elements. *Nat. Rev. Genet.* 8 973–982. 10.1038/nrg2165 17984973

[B49] Wickham H. (2016). *ggplot2: Elegant Graphics for Data Analysis*, 2nd Edn. New York, NY: Springer-Verlag.

