# Corrigendum: Genome Size Doubling Arises From the Differential Repetitive DNA Dynamics in the Genus *Heloniopsis* (Melanthiaceae)

**DOI:** 10.3389/fgene.2021.799661

**Published:** 2021-11-03

**Authors:** Jaume Pellicer, Pol Fernández, Michael F. Fay, Ester Michálková, Ilia J. Leitch

**Affiliations:** ^1^ Institut Botànic de Barcelona (IBB, CSIC-Ajuntament de Barcelona), Barcelona, Spain; ^2^ Royal Botanic Gardens, Kew, Richmond, United Kingdom; ^3^ School of Plant Biology, University of Western Australia, Crawley, WA, Australia; ^4^ Department of Botany and Zoology, Faculty of Science, Masaryk University, Brno, Czechia

**Keywords:** C-value, DNA repeats, chromosome, transposable elements, satellite DNA

In the published article, there was an error in **affiliation 4**. Instead of “**Faculty of Sciences, University of Masaryk, Brno, Czechia**”, it should be “**Department of Botany and Zoology, Faculty of Science, Masaryk University, Brno, Czechia**”.

The authors apologize for this error and state that this does not change the scientific conclusions of the article in any way. The original article has been updated.

