# Supplementary material for: Genome Size Doubling Arises From the Differential Repetitive DNA Dynamics in the Genus Heloniopsis (Melanthiaceae)
Source: Front Genet. 2021 Sep 6;12:726211. doi: 10.3389/fgene.2021.726211 (PMC8450539; doi:10.3389/fgene.2021.726211)
Supplement: Supplementary Online Resource 1 — Top four-most abundant monomers of HeloSAT. [file Table_1.DOCX]

**ONLINE RESOURCES**

**Online Resource 1.** Top four-most abundant monomers of HeloSAT.

| **Contigs** | **Length (bp)** | **Monomer length** | **Consensus sequence** | **Variants** | **A+T content (%)** |
| --- | --- | --- | --- | --- | --- |
| **CL1_11** | 338 | 26 | TCTGCTCAAGGCAACCAAGTCCTTGA | 1 | 50.0 |
| **CL1_19** | 364 | 52 | AGGCAACCAAGTCCTTGATCTGCTCAAGGCAACCAAGTCCTTGATCTGCTCA | 2 | 50.0 |
| **CL1_15** | 364 | 52 | GTCCTTGATCTGCTCAAGGTAACCAAGTCCTTGATCTGCTCAAGGCAACCAA | 3 | 48.07 |
| **CL1_23** | 390 | 78 | TCTGCTCAAGGCAACCAAGTCCTTGATCTGCTCAAGGTAACCAAGTCCTTGATCTGCTCAAGGCAACCAAGTCCTTGA | 28 | 48.71 |
