# Supplementary figures and images for: Genome Size Doubling Arises From the Differential Repetitive DNA Dynamics in the Genus Heloniopsis (Melanthiaceae)
Source: Front Genet. 2021 Sep 6;12:726211. doi: 10.3389/fgene.2021.726211 (PMC8450539; doi:10.3389/fgene.2021.726211)

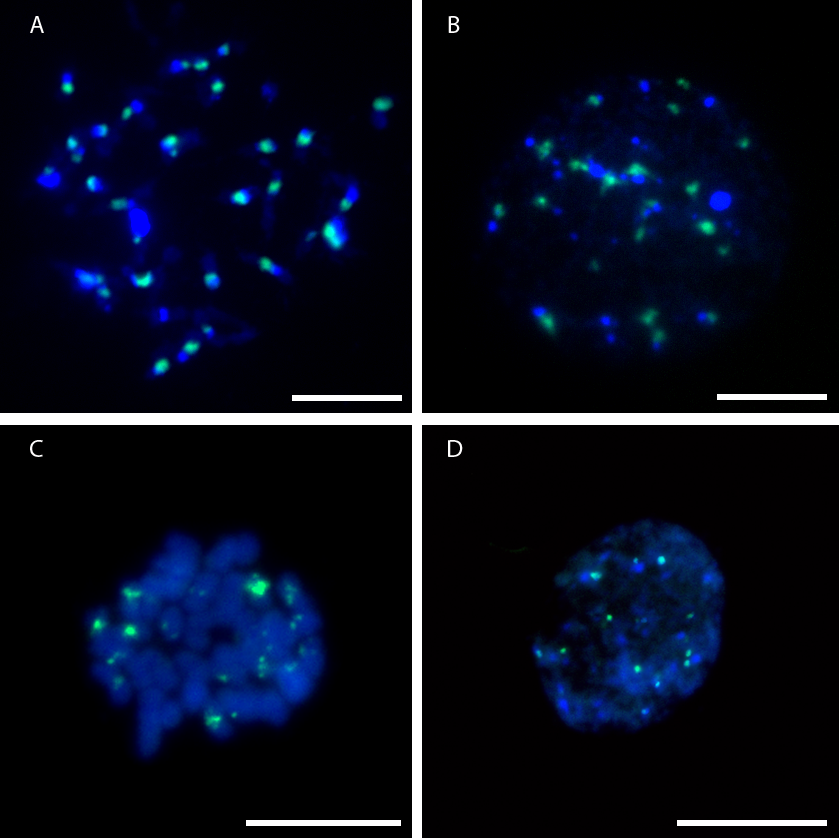

Supplement: Supplementary Online Resource 2 — Physical mapping of the satellite DNA (HeloSAT) on chromosomes and interphase nuclei of Heloniopsis umbellata (A,B) and H. koreana (C,D). [file Image_1.tif]
